# The Late Sir James Paget

**Published:** 1900-01-06

**Authors:** 


### THE LATE SIR JAMES PAGET.

ALMOST as the old year died, and as the last of the eighteen hundreds passed away, there was gathered to his fathers one who has been a man of note throughout the professional lives of all except the oldest of us. Sir James Paget was born on January 11th, 1814, at Great Yarmouth, where his father, Mr. Samuel Paget, was a merchant, and he died on the evening of December 30th, 1899, at his residence at Regent's Park. For a couple of years past he had been ailing, but he had been out of doors as lately as Christmas Day.

From the time when Paget entered as a student at St. Bartholomew's Hospital his career was attended with more than ordinary success; but it was also marked beyond the ordinary by attributes which lead to success—intelligence and industry. Intelligence he had beyond what falls to the lot of the average man—an orderly mind—but it was not by his intellectual gifts alone that Paget won fame and position, and became the trusted adviser of both high and low. It was largely to his industry, his constantly laborious industry, that Paget owed his success. His capacity for work was immense, and by concentrating his labours to a large extent upon subjects connected with his profession he attained a width and completeness of knowledge which was surprising to those who knew how busy was his life. It is a curious lesson to the more flippant among us that this man, whose fate it was to live so long, had always found it worth his while to use up every moment of his time. To those outside the profession, and outside the circle of his patients, Sir James Paget was perhaps best known as an extremely graceful speaker. Even on ordinary occasions he was a persuasive talker, but he was also what was less common, a grammatical speaker, and in his oration his sentences were models of construction—neat, like his handwriting, which is saying much. In the preparation of his speeches, moreover, he displayed the same untiring industry which in earlier life characterised his pathological studies. Not only were they prepared with the greatest care, but some of them were written out beforehand, so that he would sometimes speak for an hour without a moment's uncertainty or hesitation, never missing a point, never seeming at a loss for a word, and always choosing the word most appropriate for the idea to be expressed. Thus even in public speaking, which so obviously came easy to him, he was, as in all else, painstaking and laborious. It was thus—in dealing with his fellows—whether as a lecturer, an orator, or a consultant, that Sir James Paget was really at his best. He was one of the kindest and most judicious of friends. If he differed on any point he was ever ready to state the grounds on which his views were based, and to modify them if those grounds were shown to be faulty. To his friends he was ever staunch, and no sacrifice or trouble was too great providing he could thereby help a comrade, or bear emphatic testimony to his appreciation of public service rendered by another. Sir James Paget and Sir Richard Quain both lived to a green old age, and their professional careers illustrate some of the chief factors in the life of a consultant in London now and formerly. Nowadays surgical operations have increased in number to such an extent that the younger men have abundance of work, and it has come to pass that the public favour, on the whole, the young rather than the senior operator. Sir James Paget, though universally recognised as a master in surgery, found his patients fall off year by year from the time he gave up his active work as surgeon to St. Bartholomew's Hospital. Sir Richard Quain, on the other hand, had his consulting rooms filled almost to the last, a fact which tends to show that the consulting physician, unlike the consulting surgeon, is largely independent of his hospital connection, and may, if he chooses, carry on a large consulting practice for many years after he ceases to visit the hospitals. The private practice of a consulting surgeon nowadays must begin earlier, and will tend to decrease very often before he relinquishes active hospital work. The lives of Sir James Paget and Sir Richard Quain incidentally illustrate the truth of this contention.

Sir James Paget, who had been surgeon to the Prince of Wales since 1863, was created a baronet in 1871. He was a Fellow of the Royal Society, and was one of those appointed a Fellow of the Royal College of Surgeons when that order came into existence in 1843; he had also been president of the College.

